# Novel imaging techniques in refractory pituitary adenomas

**DOI:** 10.1007/s11102-023-01304-9

**Published:** 2023-03-27

**Authors:** J MacFarlane, KA Huynh, AS Powlson, AG Kolias, RJ Mannion, DJ Scoffings, IA Mendichovszky, HK Cheow, WA Bashari, J Jones, D Gillett, O Koulouri, Mark Gurnell

**Affiliations:** 1 Cambridge Endocrine Molecular Imaging Group, Metabolic Research Laboratories, Wellcome-MRC Institute of Metabolic Science, University of Cambridge and National Institute for Health Research Cambridge Biomedical Research Centre, Addenbrooke’s Hospital, Cambridge Biomedical Campus, Cambridge, CB2 0QQ UK; 2 Department of Neurosurgery, University of Cambridge and National Institute for Health Research Cambridge Biomedical Research Centre, Addenbrooke’s Hospital, Cambridge Biomedical Campus, Cambridge, CB2 0QQ UK; 3 Department of Radiology, University of Cambridge and National Institute for Health Research Cambridge Biomedical Research Centre, Addenbrooke’s Hospital, Cambridge Biomedical Campus, Cambridge, CB2 0QQ UK; 4 Department of Neurosurgery, Addenbrooke’s Hospital, Cambridge Biomedical Campus, Cambridge, CB2 0QQ UK; 5 Department of Nuclear Medicine, Addenbrooke’s Hospital, Cambridge Biomedical Campus, Cambridge, CB2 0QQ UK; 6 Department of Radiology, Addenbrooke’s Hospital, Cambridge Biomedical Campus, Cambridge, CB2 0QQ UK

**Keywords:** Refractory pituitary adenoma, MRI, Molecular / functional imaging, PET, Radiomics

## Abstract

Accurate localization of the site(s) of active disease is key to informing decision-making in the management of refractory pituitary adenomas when autonomous hormone secretion and/or continued tumor growth challenge conventional therapeutic approaches. In this context, the use of non-standard MR sequences, alternative post-acquisition image processing, or molecular (functional) imaging may provide valuable additional information to inform patient management.

## Introduction

Most pituitary adenomas (PA) respond to one or more conventional treatment options (e.g. medical therapy, surgery, radiotherapy) or can be managed with expectant surveillance [1]. However, some PA are refractory to standard treatment and require alternative therapeutic approaches. Refractory tumor behavior can be considered in two domains which may co-exist: (i) ‘biochemical refractoriness’ (i.e. persistent autonomous hormone production with associated clinical sequelae); (ii) ‘structural refractoriness’ [where there is continued growth (or failure to regress) with local mass effect].

Individual patient characteristics, comorbidities, genetic context, degree of endocrine dysfunction, and intraoperative and pathological findings all contribute to the decision-making process in such cases. However, in many patients imaging remains the major determinant of whether surgery and/or radiotherapy can be offered.

Standard MR sequences [coronal and sagittal T1-weighted (T1) spin echo (SE) pre- and post-gadolinium, and coronal T2-weighted (T2) fast/turbo spin echo (FSE/TSE)] provide sufficient information to guide treatment decisions in most patients [2]. However, in an important subgroup, including some refractory PA, conventional imaging is indeterminate and this may hinder optimal management.

## Additional approaches to imaging in refractory disease

To address this shortfall, several groups have explored different anatomical and molecular imaging approaches.

### Anatomical imaging

Residual tumor is most likely to localize to sites of known disease and subtle changes may only be appreciated retrospectively on review of serial imaging by a specialist multidisciplinary team.

Higher field strength MRI [3 or 7 Tesla (3T/7T)] and more advanced volumetric sequences such as 3D gradient echo (GE; e.g. spoiled and ultra-fast GE) and 3D FSE sequences may be useful for detecting small volume recurrence due to superior resolution and improved signal-to-noise and contrast-to-noise ratios. Volumetric acquisition provides improved soft tissue contrast and aids discrimination between residual adenoma, postoperative tissue remodeling and normal gland, and allows high resolution formats in any plane to guide surgery or radiotherapy planning.

Depending on the clinical question at hand, other MR sequences can be considered: [3]

- Enhanced visualization of cavernous sinus structures
  - Contrast enhanced magnetic resonance angiography (CE-MRA) uses gadolinium-based contrast agents and rapid 3D T1 spoiled GE sequences to provide enhanced delineation of intracavernous arterial anatomy.
  - High contrast and resolution T2 GE sequences (e.g. CISS) provide improved delineation of cranial nerves and the normal structure of the cavernous sinus [4].
- Improved delineation of visual pathways
  - High contrast and resolution T2 GE sequences (e.g. FIESTA) aid localization of the anterior visual pathways within the suprasellar cistern; higher signal intensity may predict persistent visual deficit [5].
  - Diffusion tensor imaging (DTI), which uses anisotropic diffusion to estimate axonal organization, may identify edema and microstructural damage within the anterior visual pathways.
- Preoperative assessment of tumor consistency
  - Magnetic resonance elastography (MRE) uses the propagation of shear waves to generate quantitative maps of tissue stiffness.
  - The apparent diffusion coefficient (ADC), calculated from diffusion-weighted imaging (DWI), may also predict tumor consistency.
- Prediction of treatment outcome
  - Although not widely employed, there is some evidence that metabolite peaks demonstrated on magnetic resonance spectroscopy (MRS) can predict the proliferative (and haemorrhagic) potential of pituitary adenomas and response to somatostatin receptor ligands in growth-hormone secreting adenomas.

### MR-based radiomics

Radiomics is the post-acquisition extraction and analysis of quantitative features from imaging datasets by means of advanced mathematical processing. It has been proposed as an adjunctive tool for characterizing and predicting the behavior of PA. Radiomic signatures from MR sequences have been shown to correlate with tumor invasiveness, pathological features (e.g. granulation pattern, Ki-67 index, transcription factor expression) and treatment responsiveness in certain tumor subtypes [6]. It may also provide an indication of the likelihood of recurrence after surgical resection [7]. Together, these preliminary findings indicate that radiomics could play a role in the identification of likely refractory PA, thereby supporting decision-making by the pituitary multidisciplinary team.

### Molecular (PET) imaging

Although an array of radiotracers have been used to image pituitary tumors, those targeting the amino acid transporter LAT1 [e.g. ^11^ C-methionine (^11^ C-Met); ^18^ F-fluoro-ethyl-tyrosine (^18^ F-FET)] or tumoral somatostatin receptor expression (e.g. ^68^Ga-DOTATATE) appear to have the greatest utility in clinical practice [8]. Coregistration of PET/CT with volumetric MRI (PET/MR^CR^), or use of hybrid PET/MR, enables precise localization of sites of abnormal tracer uptake.

#### Potential applications of molecular imaging in refractory PA

1. **Tumor localization to facilitate targeted intervention in medically refractory disease﻿.**

**Fig. 1:**
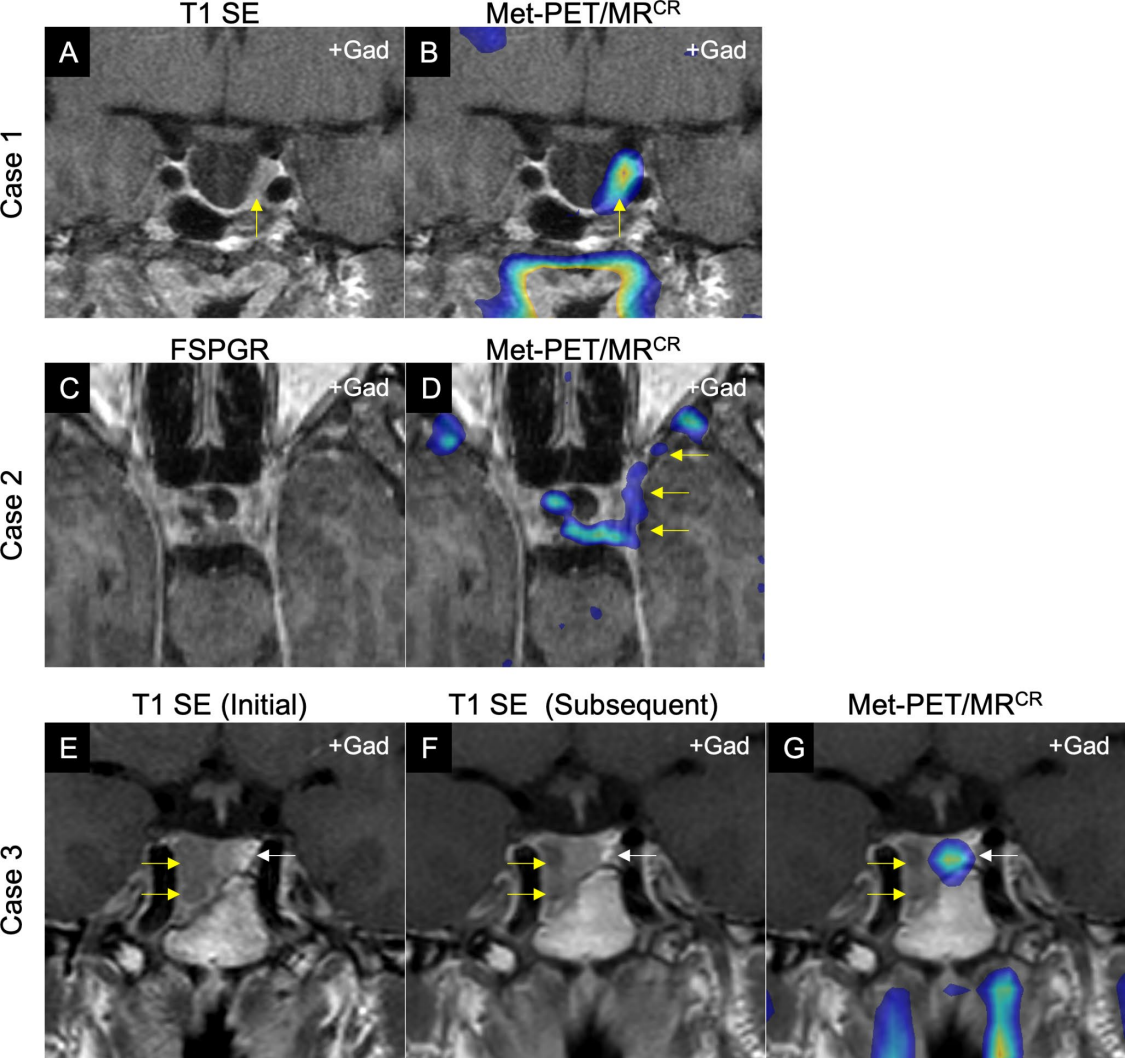
Illustrative cases demonstrating potential utility of molecular (PET) imaging in refractory pituitary adenomas.** A-B, Case 1**: Middle-aged man with acromegaly in whom initial imaging demonstrated a largely empty sella (IGF-1 3.6 ×ULN). Following primary medical therapy with maximum dose first generation somatostatin receptor ligand, the patient remained symptomatic with continued IGF-1 elevation (IGF-1 2.6 ×ULN). **A**: Post-contrast coronal T1 SE MRI showing a largely empty sella, but with an area suspicious for residual tumor adjacent to the left cavernous sinus (yellow arrow). **B**: Met-PET/MR^CR^ reveals focal tracer uptake at this site only (yellow arrow). At subsequent transsphenoidal surgery this tissue was resected, and histology confirmed a sparsely granulated somatotroph adenoma. Postoperatively, IGF-1 fully normalized, and the patient had complete resolution of symptoms. **C–D, Case 2**: Young woman with persistent Cushing disease following initial presentation with pituitary apoplexy in a 2.5 cm macroadenoma. **C**: FSPGR (volumetric) MRI in axial plane showing indeterminate appearances. **D**: Met-PET/MR^CR^ reveals significant parasellar disease not initially suspected on MRI, with tracer uptake extending along the lateral wall of the cavernous sinus towards the orbital apex (yellow arrows). **E–F, Case 3**: Middle-aged man with macroprolactinoma [presenting serum prolactin 552 ng/mL (RR 4–15)] in whom serial MRI showed only minimal reduction in tumor size following cabergoline therapy, despite complete normalization of serum prolactin (6.6 ng/mL). **E**: Post-contrast coronal T1 SE MRI at presentation demonstrating a predominantly right sided macroadenoma with infrasellar extension (yellow arrow). **F**: Repeat MRI at 24 months showing minimal tumor shrinkage (yellow arrow). **G**: Met-PET/MR^CR^ reveals only physiological tracer uptake by the normal gland in the left side of the sella (white arrow) and confirming absence of tumor activity. Key: FSPGR, fast spoiled gradient recalled echo; Met-PET/MR^CR^, 11 C-methionine PET/CT coregistered with FSPGR MRI; MRI, magnetic resonance imaging; SE, spin echo; T1, T1-weighted MRI.

MRI does not always confidently identify site(s) of small volume residual/recurrent PA, or distinguish active tumor from post-treatment remodeling. Molecular imaging can facilitate precise localization of functioning tumor to guide targeted neurosurgical resection and/or stereotactic radiosurgery (Fig. 1A–B) [9–11].

2) **Prediction of response to therapy.**

Radiotracers targeting dopaminergic (D2) receptors (e.g. ^11^ C-raclopride; ^18^ F-fallypride) may predict future responsiveness of prolactinomas and non-functioning pituitary adenomas to dopamine agonists, thereby facilitating more timely identification of tumors that are likely to be refractory to medical therapy, and allowing other treatment modalities to be considered at an earlier stage [12, 13].

Peptide Receptor Radionuclide Therapy (PRRT), typically delivered using radiolabeled somatostatin receptor ligands (e.g. ^177^Lu-DOTATATE), may be considered in the management of aggressive pituitary adenomas that have proved resistant to other treatment modalities; in this setting, molecular imaging using ^68^Ga-DOTATATE can be used to assess potential suitability for treatment [14, 15].

3) **Confirmation of boundaries/extent of anatomically complex disease.**

PA may invade local structures and extend beyond the sella, with consequences for surgical resection and radiotherapy planning. Failure to appreciate the full extent of tumor extension/invasion can lead to unanticipated non-curative intervention, creating the erroneous impression of a tumor that is refractory to standard treatment. This is particularly challenging in patients with lateral (cavernous sinus) [16] and inferior (sphenoid/clival) [17] extension where MRI appearances may appear discordant with intraoperative findings, which can be further exacerbated in the post-surgical setting. Optimized imaging with PET may reveal the true extent of tumor, thereby improving the efficacy of targeted interventions (e.g. surgery or stereotactic radiosurgery) and avoiding the misclassification of an adenoma as refractory. Equally, molecular imaging may suggest a role for treatment modalities that would otherwise have been discounted [9–11].

4) **Investigation of apparent discordant biochemical and radiological responses (‘pseudo-refractory tumors’).**

It is well recognized that some medically treated tumors (e.g. macroprolactinomas receiving primary dopamine agonist therapy) demonstrate discordant biochemical and radiological responses, most commonly achieving endocrine remission but with little to no tumor shrinkage (Fig. 1E–F). Prolactinoma guidelines have traditionally recommended treatment goals that include both a normal serum prolactin level and a 50% reduction in tumor size [18]. However, whether failure to achieve this degree of tumor shrinkage constitutes true refractoriness is debated, and in some instances delayed shrinkage is recognized even without dopamine agonist dose escalation. Molecular imaging may offer an opportunity to identify such tumors at an early stage and potentially avoid unnecessary dose titration (Fig. 1G).

## Conclusions

A small but important subgroup of PA is refractory to conventional treatment and presents a particular challenge with continued endocrine hyperfunction and/or failure of tumor regression despite multimodal therapy. Standard pituitary imaging does not always reliably identify residual tumor and this can further hinder optimal management when further treatment options are being considered. In this context, alternative MR sequences may offer additional insight regarding the extent of disease and other tumor characteristics relevant for treatment planning. The use of radiomics offers a potentially novel approach to identifying refractory tumors. Finally, we have discussed four clinical scenarios in which molecular imaging can significantly augment the effectiveness and timeliness of decision-making by accurately localizing sites of disease activity.

## Data Availability

Not applicable.
